# Economic evaluations of alcohol pharmacotherapy: Systematic review of economic evaluations of pharmacotherapy for the treatment of alcohol use disorder

**DOI:** 10.1177/00048674231201541

**Published:** 2023-10-12

**Authors:** Benjamin Higginbotham, Joahna Kevin Perez, Eva Louie, Paul S Haber, Dan Lubman, Shalini Arunogiri, Mary Lou Chatterton, Kirsten C Morley

**Affiliations:** 1 Specialty of Addiction Medicine, Faculty of Medicine and Health, The University of Sydney, Camperdown, NSW, Australia; 2 Public Health and Preventive Medicine, Monash University, Melbourne, VIC, Australia; 3 Monash Addiction Research Centre and Eastern Health Clinical School, Monash University, Melbourne, VIC Australia

**Keywords:** Alcohol use disorder, pharmacotherapy, economic evaluation, systematic review

## Abstract

**Objective:**

Alcohol use disorders confer a significant burden of disease and economic cost worldwide. However, the utilisation of pharmacotherapies to manage alcohol use disorder is poor. We aimed to conduct a systematic review of economic evaluation studies of alcohol use disorder pharmacotherapies.

**Methods:**

A search was conducted in Embase, Medline, CINAHL, PsychINFO and EconLit (August 2019, updated September 2022). Full economic evaluations using pharmacotherapy to treat alcohol use disorders were included. Included studies were stratified by medication and summarised descriptively. The Consensus on Health Economic Criteria list was used to assess the methodological quality.

**Results:**

A total of 1139 studies were retrieved, of which 15 met the inclusion criteria. All studies were conducted in high-income countries. Four studies analysed nalmefene, four studies assessed acamprosate, three for naltrexone and four for stand-alone and/or combinations of naltrexone and acamprosate. There were 21 interventions synthesised from 15 studies as some studies evaluated multiple interventions and comparators. More than half of the included studies (73%) reported pharmacotherapy as dominant (less costly and more effective than comparators). From healthcare payer perspectives, five studies found that pharmacotherapy added to psychosocial support was dominant or cost-effective, accruing additional benefits at a higher cost but under accepted willingness to pay thresholds. Three analyses from a societal perspective found pharmacotherapy added to psychosocial support was a dominant or cost-effective strategy. Quality scores ranged from 63% to 95%.

**Conclusion:**

Pharmacotherapy added to psychosocial support was cost-effective from both healthcare and societal perspectives, emphasising an increased role for pharmacotherapy to reduce the burden of alcohol use disorders.

## Introduction

Alcohol use accounts for a significant proportion of the burden of disease, with 5.3% of all global deaths and 4.2% (99.2 million) disability-adjusted life years (DALYs) attributable to alcohol consumption (GBD 2016 Alcohol Collaborators, 2018). Alcohol use is a known risk factor for several diseases and conditions, including liver cirrhosis (Roerecke et al., 2019) and various cancers, including those of the colorectum, mouth, throat, liver and breast (Rehm et al., 2003). Alcohol consumption has also been linked to a range of psychiatric disorders, contributing to a considerable proportion of suicides globally (World Health Organisation [WHO], 2018). Alcohol use disorders (AUDs) rank among the most prevalent mental disorders globally and are defined as a pattern of alcohol consumption resulting in dependence symptoms and negative physical and mental health complications and social consequences (Haber et al., 2021).

Along with the harm to self and others, alcohol misuse also imposes a substantial economic burden worldwide (1–3). Previous research indicates a range of estimates including 1.3–3.3% of total health costs, 6.4–14.4% of total public order and safety costs, 0.3–1.4 per thousand of gross domestic product (GDP) for criminal damage costs, 1.0–1.7 per thousand of GDP for drink-driving costs, and 2.7–10.9 per thousand of GDP for work-place costs (absenteeism, unemployment and premature mortality) (Baumberg, 2006). Tangible costs associated with the provision of healthcare for alcohol use are significant; however, the majority of costs are incurred outside of the healthcare system including lost productivity, road accidents and criminal justice system costs (Manning et al., 2013). Intangible costs such as premature mortality and impaired quality of life far exceed tangible costs (Whetton et al., 2021). To reduce these financial costs, in addition to alcohol’s impact on the burden of disease, it is crucial to implement effective interventions that are targeted at the segment of the population that are not responding to public health measures and are continuing to drink excessively.

Several pharmacotherapies are emerging for the management of AUD (Morley et al., 2021). At present, the pharmacological treatments specifically indicated for alcohol use disorders are disulfiram, acamprosate, naltrexone and nalmefene. However, unfortunately, these medications are under-utilised, with very low rates of uptake being reported across the world including in the UK (Donoghue, 2021), USA (Mark et al., 2003), Australia (Morley et al., 2016, 2017) and Europe (Holzbach et al., 2019). Low uptake rates are also observed in primary care settings (Thompson et al., 2017) and in the community, as is observed in Australia where only 2.7% of individuals estimated to have AUD are prescribed medications outside of hospital settings (Morley et al., 2016, 2017). These are alarming figures, particularly given the existence of clear recommendations supporting pharmacological interventions in national guidelines across the globe (Haber et al., 2021; National Institute for Health and Care Excellence (NICE), 2011), multiple meta-analyses indicating efficacy in reducing consumption (Jonas et al., 2014) and data indicating prescribing alcohol pharmacotherapies is associated with lower risk of hospitalisation compared with no uptake of any alcohol pharmacotherapy (Heikkinen et al., 2021).

To this degree, in a public healthcare system with a limited budget, consideration of economic factors related to treatments is essential to efficiently allocate resources. Full economic evaluations explicitly compare the costs and effectiveness of a health intervention to a comparison intervention (Turner et al., 2021). Evaluations report the incremental cost-effectiveness ratio (*ICER)*, which is expressed as the ratio of the difference in costs between two strategies to the difference in effectiveness (Paulden, 2020) in addition to the willingness-to-pay (WTP) threshold which is the maximum price which a healthcare consumer might pay for health benefit (Turner et al., 2021).

Unfortunately, there is a scarcity of such studies that synthesise the health economic literature for alcohol pharmacotherapies. There has been one review which evaluated published data on the cost-effectiveness of acamprosate (Poldrugo et al., 2005). This review indicated that acamprosate was dominant over other rehabilitation strategies. However, since this review was conducted, there has been increased use of naltrexone (Haber et al., 2021) and there have also been further economic evaluations. A more recent narrative review summarised the cost-effectiveness literature, including pharmacological and psychosocial therapies (Rehm and Barbosa, 2018). These authors concluded that many studies pointed towards existing cost-beneficial therapies overall but that research was scarce. However, this review was not specific to pharmacotherapy and did not include the assessment of study quality. Accordingly, to address these gaps, the current study aimed to provide a comprehensive and up-to-date systematic review of economic evaluations relating to pharmacologic treatment of AUDs.

## Methods

This systematic review (pharmacotherapy for alcohol use disorder) was a component of the original method registered on the International Prospective Register of Systematic Reviews (PROSPERO) database under ID CRD42020147403 (28 April 2020).

### Search strategy

A comprehensive electronic literature search was conducted across multiple databases, including Embase, Medline, CINAHL, PsychINFO and EconLit in August 2019 and was updated in September 2022, to ensure broad coverage of relevant biomedical, medical, psychological and economic literature (see Supplementary material). Bibliographies of relevant articles were also hand-searched to identify potentially eligible studies that were not identified in the electronic search. Terms relating to economic evaluation, alcohol use disorder and pharmacologic treatment were used to search for relevant literature. Searches were further refined to include peer-reviewed journals in English only, and no restriction on the year of publication was used.

### Selection criteria

All studies were imported into Covidence where duplicates were identified and removed. Titles and abstracts were screened independently by four reviewers (BH, EL, JP and MLC), and relevant full-text articles were obtained and assessed against the inclusion criteria. Disagreements across the screening process were resolved by discussion among all authors until consensus was achieved.

We focussed on those articles where alcohol was the primary drug of concern and treatment involved pharmacotherapy. Studies were included if they were full economic evaluations, comparing costs and effectiveness of at least two interventions for AUD. Studies that examined patient populations with significant mental health only or other addiction comorbidities (except nicotine) were excluded given different management strategies. Cost studies, reviews, study protocols, dissertations, book chapters, expert opinions, conference papers and non-English publications were also excluded from this review.

### Data extraction

Data extraction was adapted from the review guidelines for economic evaluations developed by Joanna Briggs Institute (JBI) (Gomersall et al., 2015) and was carried out in Microsoft Excel by two reviewers (BH and JP) and checked by a third reviewer (MLC). Included studies were categorised according to the type of treatment evaluated. There was heterogeneity present among included studies; therefore, data were synthesised narratively and in tabular format. Extracted data items were authors, year and country of publication, study population, type of study, type of economic evaluation, perspective and time horizon, reference year, discount rate and currency, cost categories, outcome measures, results, and where applicable, ICERs. For comparison across studies, cost values were converted to 2022 Australian dollars (A$) using The Campbell and Cochrane Economics Methods Group and the Evidence for Policy and Practice Information Coordinating Centre (CCEMG-EPPI-Centre) cost converter (Shemilt et al., 2010). For studies not reporting a reference year for costs, an assumption was made to use the year the trial was conducted or two years prior to the publication, if the trial year was not reported.

### Synthesis of findings

A narrative synthesis was conducted along with the dominance ranking framework that has been previously used in the literature and was adapted from a systematic review of economic evaluation guidelines developed by JBI (Gomersall et al., 2015). The framework presents the distribution of interventions based on costs and effectiveness across three decision criteria: favour intervention, unclear or reject intervention. A traffic light colour coding was used to indicate implications for decision-makers. A ‘green’ segment indicates that the result of the study suggests that the intervention is likely to be favoured and accepted by decision-makers (i.e. dominant ICERs, lower costs and better outcomes). A ‘yellow’ segment shows that the study’s intervention does not result in a clear-cut decision context. For instance, the intervention is more effective but also more costly, or a value for money threshold to determine whether the intervention is cost-effective was not reported. Finally, a ‘red’ segment suggests that the routine adoption of the intervention being studied is likely to be less favoured or rejected by decision-makers (i.e. dominated ICER, higher costs and less effective interventions).

### Quality assessment

Quality assessment of included economic evaluation studies was based on the Consensus on Health Economic Criteria (CHEC), a set of 19 questions with a response option of ‘yes’ if the study adequately addressed the specific criteria or ‘no’ if the criteria have not been fulfilled or inadequate information is available (Evers et al., 2005). This tool was chosen for its succinctness, reliability and high correlation with other commonly used criteria, including the BMJ checklist (Gerkens et al., 2008). Two authors (BH and JP) independently assessed the methodological quality of each paper, and any disagreement was resolved by discussion or the aid of another author (MLC). Each CHEC question was given 1 point for a ‘yes’ and 0 for a ‘no’ with a total score ranging from 0 to 19 and then converted to a percentage.

## Results

### Study selection and inclusion

**Figure 1.**
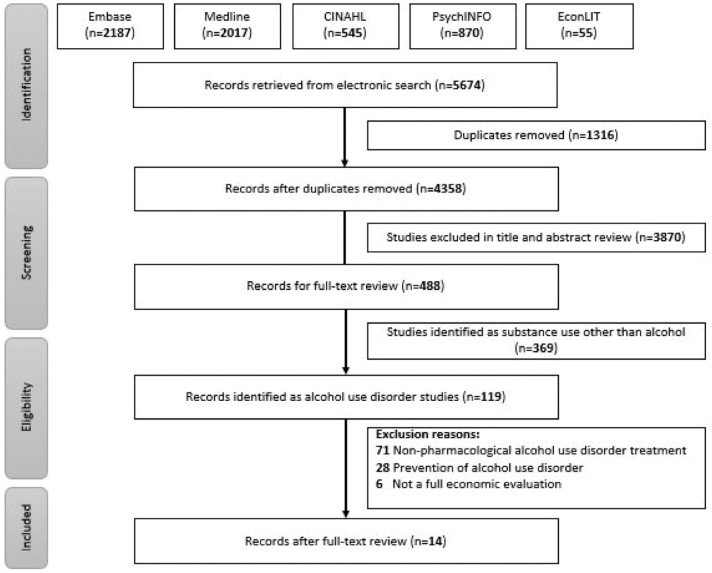
PRISMA diagram.

The updated literature search identified 1139 records that were assessed for their title and abstract after removing 290 duplicates. After screening 849 abstracts, 143 records were identified for full-text review. Finally, 15 studies met the inclusion criteria for full economic evaluations of pharmacotherapy for alcohol use disorder and were included in the review (Figure 1).

### Characteristics of included studies

Study characteristics can be viewed in Table 1. Of the 15 included studies, four assessed the effectiveness of nalmefene (Brodtkorb et al., 2016; Laramee et al., 2014, 2016; Millier et al., 2017); four evaluated the effects of acamprosate (Annemans et al., 2000; Palmer et al., 2000; Rychlik et al., 2003; Schädlich and Brecht, 1998); three assessed naltrexone (Cobiac et al., 2009; Mortimer and Segal, 2005; Walters et al., 2009); and three evaluated either stand-alone and/or a combination of naltrexone and acamprosate (Dunlap et al., 2010; Sluiter et al., 2018; Zarkin et al., 2008). Another study (Avanceña et al., 2021) focussed on US Food and Drug Administration (FDA) approved (acamprosate or naltrexone) and non-FDA-approved (baclofen, gabapentin, topiramate) medications. In total, 21 interventions were synthesised among 15 included studies, as some studies have evaluated multiple interventions and/or comparators.

**Table 1.** Details of included studies.

Lead author (year), country	Economic evaluation type	Study design	Interventions and comparator	Population description	Perspective, time horizon	Currency, year of pricing, discount rates	Cost categories	Outcomes	Results/key points/sensitivity analysis
**Nalmefene**									
Brodtkorb et al. (2016), UK	CUA	RCT	1. Nalmefene + psychosocial support (BRENDA) 2. Psychosocial support (BRENDA)	Adults with both alcohol dependence and high or very high DRLs who do not require immediate detox and who have high or very high DRLs after initial assessment	Societal, 5 years	UK Pound (£); Ref year: 2012; Discount rate: 3.5%	Medical Treatment; Drug treatment; Harmful events; Productivity loss; Crime and Justice	QALYs (EQ-5D)	Nalmefene + pyschosocial support was dominant (Q2); WTP: £20,000/QALY gained
Millier et al. (2017), UK	CUA	Modelling	1. Nalmefene + psychosocial support 2. Psychosocial support only 3. No treatment	Patients with a DRL that was consistently high/very high between baseline and randomisation	Societal perspective and TPP, 5 years	UK Pound (£); Ref year: 2011; Discount rate: 3.5%	Direct cost -Medical treatment cost -Drug treatment-related costs -Harmful events Indirect cost -Crime and justice costs -Productivity losses	-QALYs (utilities obtained from the literature) -Probabilities of alcohol-attributable diseases, injuries and deaths	TPP perspective (5 years): -NMF + PS vs PS only: ICER £10,613 per QALY gained -NMF + PS vs No treatment: ICER £7,858 per QALY gained Societal perspective: NMF + PS dominated other treatments WTP: £20,000/QALY gained
Laramee et al. (2014), UK	CUA	Modelling	1. Nalmefene + psychosocial support (BRENDA) 2. Psychosocial support only (BRENDA)	Adults with both alcohol dependence and high or very high DRLs who do not require immediate detox and who have high or very high DRLs after initial assessment	NHS in England and Wales, 5 years	UK Pound (£); Ref year: 2010/2011; Discount rate: 3.5%	Treatment costs: -Cost of drug -Cost of psychosocial support session Costs and resource use associated with occurrence of alcohol-attributable harmful events	QALYs (EQ-5D); number of alcohol-attributable harmful events avoided	-Incremental costs £434 -Incremental QALYs 0.08 -ICER = £5204/QALY gained -7179 alcohol-attributable diseases/injuries avoided per 100,000 patients -309 deaths avoided per 100,000 patients WTP: £20000/QALY gained
Laramee et al. (2016), UK	CUA	Modelling	1. Nalmefene + psychosocial support (BRENDA) 2. psychosocial support only (BRENDA)	Adults with both alcohol dependence and high or very high DRLs who do not require immediate detox and who have high or very high DRLs after initial assessment	NHS, 5 years	UK Pound (£); Ref year: 2012; Discount rate: 3.5%	Treatment-related costs: -Cost of drug -Cost of psychosocial support session Second-line treatment strategy costs: -Cost for assisted alcohol withdrawal to reach abstinence -Pharmacological cost (acamprosate or naltrexone) -Medical management cost -Psychological interventions Costs for managing alcohol-attributable harmful events	QALYs (EQ-5D)	-Incremental costs -£60 -Incremental life years gained 0.009 -Incremental QALYs 0.071 -ICER, cost/QALY – dominant WTP: £20,000/QALY gained
**Acamprosate**									
Rychlik et al. (2003), Germany	CEA	Prospective cohort study	1. Adjuvant acamprosate therapy 2. Standard psychosocial rehabilitation programme, no pharmacotherapy (standard cohort)	Patients 18–65 years who had contacted their physicians for alcohol-related problems, fulfilled *DSM*-IV criteria [1] for alcohol dependence and had been prescribed an acute alcohol detoxification, followed by rehabilitation.	Not clearly specified, 1 year	EUR; Ref year: not clearly specified (assuming 1998/1999 when the study was performed) Discount rate: 0	Direct medical costs: -Physician visits -ED -Diagnostic and laboratory tests -Drugs -Non-medical treatments -Nursing -Hospitalisations -Treatment of undesirable effects and side effects Indirect costs (economic): -Productivity losses -Travel expenses	Abstinence rate	Per-protocol analysis (PPA): 1. With acamprosate Average total costs per patient = EUR 1631.49 per year = 33.6% remained abstinent = CE: EUR 4,857.22/abstinence 2. Standard cohort Average total costs per patient = EUR 2068.83 per year = 21.1% remained abstinent = CE: EUR 9790.03/abstinence Intention-to-treat analysis (ITT): 1. With acamprosate Average total costs per patient = EUR 1591.59 per year = 32.4% remained abstinent = CE: EUR 4911.24/abstinence 2. Standard cohort Average total costs per patient = EUR 2003.87 per year = 20.4% remained abstinent = CE: EUR 9804.6 /abstinence
Schädlich and Brecht (1998), Germany	CEA	Modelling	1. Adjuvant acamprosate therapy 2. Standard psychosocial rehabilitation programme, no pharmacotherapy (standard cohort)	Patients who met at least 5 Diagnostic and Statistical Manual, third edition-revised (*DSM*-III-R) criteria for alcohol dependence and were alcohol-dependent according to the Munich Alcoholism Test	German healthcare system and Statutory Health Insurance scheme, 10 years	German Marks (DM); Ref year: 1995 Discount rate: 5%	Direct medical costs: -Cost of drug -Case-related treatment costs of the target events (hospitalisation and rehabilitation) -Relapse treatment	Abstinence rate	German Healthcare System: Acamprosate has a net savings of DM 2602 per additional abstinent patient Statutory Health Insurance Scheme: Acamprosate has a net savings of DM 530 per additional abstinent patient
Palmer et al. (2000), Germany	CEA	Modelling	1. Standard counselling therapy + 48 weeks of adjuvant Acamprosate 2. standard counselling therapy only	Male cohort with an average age of 41 years, 80% with fatty liver, 15% with cirrhosis, 22% with pancreatitis and 1% with alcoholic cardiomyopathy at baseline	German health insurance perspective, lifetime	German Marks (DM); Ref year: 1996 Discount rate: 5%	Direct medical costs: -Cost of acamprosate -Alcohol dependence syndrome (cost if Relapsed) -Costs related to complications such as: liver disease, GI disease, cardiomyopathy, alcohol psychosis or peripheral neuropathy	-Life expectancy -Mean expected total lifetime costs per patient	Life expectancy: therapy + acamprosate vs therapy only = 0.52 life years gained Average expected Lifetime costs per patient: 1. therapy + acamprosate = DEM 48,245 2. therapy only = DEM 49,907
Annemans et al. (2000), Belgium	CBA	Modelling	1. Acamprosate (Campral) 2. Placebo	Alcohol-dependent patients	Belgian health insurance perspective, 2 years	Belgian Francs (BEF); Ref year: not stated Discount rate: not stated	Direct medical costs: -Consultations -Biochemistry examinations -Drugs -Hospital costs -Complications (liver)	Average cost of maintaining abstinence	Total expected cost over 24 months per patient: Acamprosate: BEF 211,986 Placebo: BEF 233,287 Net cost-savings of BEF 21,301 per patient
**Naltrexone**									
Cobiac et al. (2009), Australia	CUA	Modelling	1. Volumetric taxation 2. Advertising bans 3. Increase in minimum legal drinking age 4. Licencing controls on operating hours 5. Brief intervention (with and without GP telemarketing and support) 6. Drink-driving campaigns 7. Random breath testing 8. Residential treatment for alcohol dependence (with and without Naltrexone); each intervention is compared to current practice	(1,2,4) 100% of population aged 18+ (3) 100% of population (drivers) aged 18–20 years (5) 2–3% of hazardous/harmful drinkers aged 18–79 years (6,7) 100% of population (drivers) aged 18+ years (8) 4% of alcohol dependents aged 18–79 years	Australian Healthcare system, Lifetime	AUD ($); Ref year: 2003; Discount rate: 3%	Intervention cost: government cost for enforcement/delivery of interventions; costs to patients (time and travel) Cost offsets: costs related to treatment of alcohol-related diseases and injuries.	DALYs	Residential treatment with Naltrexone: -Not cost-saving -Median ICER $120,000 -0% probability of being cost-effective under $50,000/DALY WTP: $50,000/DALY gained
Mortimer and Segal (2005), Australia	CUA	Modelling	1. Brief interventions for problem drinkers vs no alcohol-related treatment 2. Psychotherapy for mild-to-moderate dependence vs BSCT 3. Drug therapy (naltrexone) + counselling for detoxified patients with a history of severe physical dependence vs Placebo + counselling	1. Heavy drinkers aged > 19 years 2. Mild to moderately dependent drinkers 15–59 years 3. Recently detoxed, no coexisting drug use, no significant psych disorder	Societal perspective, Lifetime	AUD ($); Ref year: 2003; Discount rate: 5%	Not clearly stated	QALYs (utilities obtained from the literature)	3. Naltrexone + counselling vs Placebo + counselling ICER $12,966 per QALY WTP: $50,000/QALY gained
Walters et al. (2009) (Australia)	CEA and CUA	Single-centre non-RCT retrospective study	1. CBT + Naltrexone 2. CBT alone	patients at least 18 years of age, who met the *DSM*-IV criteria for alcohol dependence, had bMAST scores associated with alcohol dependence (more than 6), 29 and had maintained a minimum of 3 days abstinence and no other substance dependence (except nicotine)	not clearly specified, most likely treatment provider’s perspective, 12 weeks	AUD ($); Ref year: not stated Discount rate: 0	-Personnel costs -Supplies and materials costs -Equipment costs -Contracted services costs -Buildings and facilities costs -Miscellaneous resources	-Utilities (SF-6D) -Successful treatment outcomes (completed all 8 sessions and abstinent throughout the programme)	1. CBT + Naltrexone -SF-6D increased from pre- to post-treatmentt -62.6% remained abstinent and completed the programme -average cost = $1,241 per patient 2. CBT only -SF-6D increased from pre- to post-treatment -36.1% remained abstinent and completed the programme -average cost = $739 per patient *No difference in utilities between groups *Weak dominance for (1) CBT + Naltrexone
**Naltrexone and/or acamprosate**									
Dunlap et al. (2010), USA	CEA	RCT	1. Medical Management (MM) + placebo 2. MM + Naltrexone 3. MM + Naltrexone +Acamprosate 4. Combined behavioural intervention (CBI) only 5. MM + Acamprosate 6. MM + placebo + CBI 7. MM + Naltrexone + CBI 8. MM + Naltrexone +Acamprosate + CBI 9. MM + Acamprosate + CBI	Patients with diagnoses of primary alcohol dependence	Patient perspective, 16 weeks	US Dollars ($); Ref year: 2007 Discount rates: 0	-Treatment cost -Medication cost (Copays) -Office visits cost (health prof) (Copays) -Assessment time cost -Session time cost -Self-help programme time cost -Travel cost	-Proportion of patients with good clinical outcomes (abstinent, or moderate drinking without problems) -Proportion of patients that do not return to heavy drinking -Percent days abstinent from alcohol	ICER (2) MM + Naltrexone VS (1) MM + placebo = $575.19 per patient achieving a good clinical outcome = $1022.56 per patient avoiding a return to heavy drinking = $14.84 per patient for a percentage point increase in percent days abstinent (3) MM + Naltrexone + Acamprosate VS (2) MM + Naltrexone = $1242.75 per patient achieving a good clinical outcome =$1242.75 per patient avoiding a return to heavy drinking = $99.42 per patient for a percentage point increase in percent days abstinent *MM + Acamprosate and all other interventions that include CBI were economically dominated by interventions 1–3.
Zarkin et al. (2008), USA	CEA	RCT	1. Medical Management (MM) + placebo 2. MM + Naltrexone 3. MM + Naltrexone +Acamprosate 4. Combined behavioural intervention (CBI) only 5. MM + Acamprosate 6. MM + placebo + CBI 7. MM + Naltrexone + CBI 8. MM + Naltrexone +Acamprosate + CBI 9. MM + Acamprosate + CBI	1. Alcohol dependence as determined by *DSM*-IV12 criteria; 2. 4 to 21 days of abstinence; and 3. more than 14 drinks per week for women or 21 drinks per week for men, with at least 2 heavy-drinking days, defined as 4 drinks per day for women and 5 drinks per day for men, during a consecutive 30-day period within the 90 days before baseline evaluation.	Treatment provider, 16 weeks	USD ($); REF YEAR: 2007 DISCOUNT RATE: 0	-Medication costs -Labour for MM -Labour for CBI -Laboratory and non-laboratory assessments	-Percent days abstinent -Proportion of patients who avoid heavy drinking -Proportion of patients with good clinical outcome	Percent days abstinent: -MM + Naltrexone vs MM + Placebo = ICER $42.24 -MM + Naltrexone + Acamprosate vs MM + Naltrexone = ICER $663.80 Proportion of patients who avoid heavy drinking: -MM + Naltrexone vs MM + Placebo = ICER $2,846.85 -MM + Naltrexone + Acamprosate vs MM + Naltrexone = ICER $8,095.12 Proportion of patients with good clinical outcomes: -MM + Naltrexone vs MM + Placebo = ICER $1,689.74 -MM + Naltrexone + Acamprosate vs MM + naltrexone = ICER $7543 *MM + Acamprosate and all other interventions that include CBI were economically dominated by interventions 1–3.
Sluiter et al. (2018), Netherlands	CUA	Modelling	1. Genotype-guided treatment (G-allele carriers receiving naltrexone; AA homozygotes acamprosate or naltrexone) 2. Standard care (random treatment allocation to acamprosate or naltrexone)	Alcohol use disorder patients	Societal perspective, 1 year	EUR; Ref year: 2015 Discount rate: 0	-Healthcare costs -Non-healthcare costs -Indirect costs	QALYs (utilities obtained from the literature)	Incremental cost = EUR 66 Incremental QLAYs = 0.005 ICER = EUR 13,350 per QALY 75% probability of being cost-effective at EUR 80,000/QALY threshold WTP: EUR 80,000/QALY gained
Avancena et al. (2021), USA	CUA	Modelling	1. FDA-approved Medication-assisted therapies (MATS) (acamprosate and naltrexone) 2. Non-FDA-approved MATS (baclofen, gabapentin, topiramate) 3. Counselling 4. Do nothing	Hypothetical cohort of 54-year-old patients with compensated AC	Healthcare and societal perspective, Lifetime	US Dollars ($); Ref year: 2017 Discount rates: 3%	Intervention costs: -Drug costs -Individual alcohol cessation counselling costs Medical management costs: -Compensated and decompensated AC treatment -Hepatocellular carcinoma treatment -Liver transplantation and treatment-related costs Lifetime productivity costs Health and consumption costs	QALYs (utilities obtained from the literature)	Healthcare perspective: -FDA-approved MATS vs Do-nothing = cost-saving -non-FDA-approved MATS vs Do-nothing = cost-saving -Counselling vs Do-nothing = cost-saving Societal perspective: -FDA-approved MATS vs Do-nothing = cost-saving -non-FDA-approved MATS vs Do-nothing = cost-saving -Counselling vs Do-nothing = $3,724 per QALY ICER compared to the next most expensive undominated option: -FDA-approved MATS (acamprosate, naltrexone) were dominant from both societal and healthcare perspectives WTP: $50,000 and $100,000/QALY gained

AA: alcoholics anonymous; AC: alcohol-related cirrhosis; AUD: alcohol use disorders; BRENDA: A psychosocial program with 6 components including: 1) a biopsychosocial evaluation; 2) a report of findings from the evaluation given to the patient; 3) empathy; 4) addressing patient needs; 5) providing direct advice; and 6) assessing patient reaction to advice and adjusting the treatment plan as needed; BSCT: behavioural self-control training; CBA: cost-benefit analysis; CBT: cognitive behavioural therapy; CE: cost effectiveness; CEA: cost-effectiveness analysis; CUA: cost-utility analysis; DALY: disability-adjusted life years; DEM: german marks; DRLs: drinking-risk levels; ED: emergency department; EUR: euro; FDA: Food and Drug Administration; GI: gastrointestinal disease; GP: general practice; ICER: incremental cost-effectiveness ratio; NHS: National Health Service; NMF: nalmefene plus; MM: medical management; PS: psychosocial support; QALY: quality-adjusted life year; RCT: randomised controlled trial; TPP: third-party payer; USD: US dollar; WTP: willingness to pay.

The included studies that used three types of economic evaluation, 8 (53%) used a cost-utility analysis (CUA), 5 (33%) conducted a cost-effectiveness analysis (CEA), 1 (6%) study used both CUA and CEA and 1 (6%) conducted a cost-benefit analysis (CBA). Outcomes measured in CEA studies include abstinence rate (Rychlik et al., 2003; Schädlich and Brecht, 1998); life expectancy and average expected lifetime cost (Palmer et al., 2000); the proportion of patients with good clinical outcomes and percent days abstinent (Dunlap et al., 2010; Zarkin et al., 2008). In the realm of CUA studies, where the utility instrument for deriving QALYs was reported the EQ-5D tool was frequently used (*n* = 3) (Brodtkorb et al., 2016; Laramee et al., 2014, 2016) and one study used utility derived from SF-6D (Walters et al., 2009), whereas the others derived their QALYs from the literature (Avanceña et al., 2021; Millier et al., 2017; Mortimer and Segal, 2005; Sluiter et al., 2018). The majority of the studies (*n* = 10) performed economic modelling (Annemans et al., 2000; Avanceña et al., 2021; Cobiac et al., 2009; Laramee et al., 2014, 2016; Millier et al., 2017; Mortimer and Segal, 2005; Palmer et al., 2000; Schädlich and Brecht, 1998; Sluiter et al., 2018). Three studies conducted the economic evaluation as part of a randomised controlled trial (RCT) (Brodtkorb et al., 2016; Dunlap et al., 2010; Zarkin et al., 2008), and the remaining studies (Rychlik et al., 2003; Walters et al., 2009) used observational study designs. Adults aged 18 and above with alcohol dependence were the main target population for most studies. Among these, four studies included those with high or very high drinking-risk levels (DRLs), three studies fulfilled the Diagnostic and Statistical Manual (*DSM*-IV) criteria for alcohol dependence and one study used *DSM*-III, while only one used alcohol harmful use and dependence criteria from the International Classification of Disease (ICD-10). Most of the studies (8 studies) were conducted in European countries such as the United Kingdom (UK) (Brodtkorb et al., 2016; Laramee et al., 2014, 2016; Millier et al., 2017), Germany (Palmer et al., 2000; Rychlik et al., 2003; Schädlich and Brecht, 1998), Netherlands (Sluiter et al., 2018) and Belgium (Annemans et al., 2000). The remaining studies were from Australia (Cobiac et al., 2009; Mortimer and Segal, 2005; Walters et al., 2009) and the USA (Avanceña et al., 2021; Dunlap et al., 2010; Zarkin et al., 2008).

### Main findings

#### Nalmefene

Four studies reported cost-utility analyses of nalmefene plus psychosocial support (NMF + PS). All four were conducted in the UK with a 5-year time horizon with the comparator of psychosocial support alone (PS only), and one study by Millier et al. (2017) had an additional comparator of no treatment.

The study by Brodtkorb et al. (2016) was the only RCT among the four studies and utilised a societal perspective, while a modelling study by Millier et al. (2017) was based on a societal perspective and that of a third-party payer (TPP). Costs measured for both studies included intervention costs, costs of medical treatment including harmful events, and the addition of productivity losses and costs related to crime and justice from the societal perspective. Brodtkorb et al. (2016) and Millier et al. (2017) found the NMF + PS dominant with QALYs gained and societal costs reduced. On the other hand, from the perspective of a TPP, Millier et al. (2017) found the intervention was more costly and more effective when compared to either PS only or no treatment, but produced an ICER well below the commonly used willingness to pay threshold of £20,000 per QALY gained.

Two other modelling studies found that NMF + PS was dominant (Laramee et al., 2016) and cost-effective with an ICER (£5204/QALY gained) (Laramee et al., 2014) falling below the willingness to pay threshold when compared to psychosocial support alone from the National Health Services (NHS) perspective.

#### Acamprosate

Three CEA studies all conducted in Germany and one CBA study from Belgium evaluated acamprosate therapy. Two studies modelled the effects of acamprosate (ACP) compared to a standard psychosocial rehabilitation programme (Schädlich and Brecht, 1998) and acamprosate plus standard counselling (ACP + counselling) compared to standard counselling only (Palmer et al., 2000). From a German health insurance perspective, both studies found acamprosate to be cost-saving combined with a better abstinence rate (Schädlich and Brecht, 1998) and 0.52 incremental life-years gained (Palmer et al., 2000). Schädlich and Brecht (1998) also found that even with added costs from the perspective of the German healthcare system, acamprosate was still cost-saving and improved outcomes (additional abstinent patient). Another modelled CBA study that evaluated acamprosate against a placebo from a Belgian health insurance perspective found acamprosate to be cost-saving (Annemans et al., 2000). In addition, a prospective naturalistic cohort study by Rychlik et al. (2003) compared acamprosate to a standard psychosocial rehabilitation programme. Abstinence rate was the outcome measure, and costs included direct medical and indirect costs (travel costs and productivity loss). While the study (Rychlik et al., 2003) calculated the average cost per abstinence rate for each group, standard economic evaluation techniques would evaluate the ratio of the difference in costs and outcomes between the comparison groups. Through that lens, acamprosate was dominant as the acamprosate group had lower costs ($1592 vs $2004 per patient per year) and better outcomes (32% vs 20% abstinence rate) than the standard care comparator.

Meanwhile, two studies from the US evaluated multiple interventions including acamprosate in addition to medical management (MM + ACP) and MM + ACP combined with behavioural intervention (CBI) (MM + ACP + CBI) (Dunlap et al., 2010; Zarkin et al., 2008). From the perspective of the patient Dunlap et al. (2010) and treatment provider Zarkin et al. (2008), MM + ACP, and MM + ACP + CBI were economically dominated since they had higher mean costs and lower mean effectiveness than other comparators (MM + natrexone and MM + naltrexone + ACP).

#### Naltrexone

Three studies that analysed the effects of naltrexone (NTX) were from Australia. A modelling paper by Cobiac et al. (2009) found that from the perspective of the Australian healthcare system, residential treatment with and without naltrexone was not a cost-effective programme compared with seven other prevention programmes (i.e. volumetric taxation, advertising bans). While the analysis demonstrates the trade-off between prevention and treatment, the comparator does not provide an assessment of the cost-effectiveness of naltrexone compared to standard treatment for alcohol problems. The other modelling study by Mortimer and Segal (2005) found that from a societal perspective, naltrexone plus counselling, when compared with placebo plus counselling, resulted in an ICER of $19,454 per QALY gained. Given this ICER falls well below the accepted willingness-to-pay (WTP) threshold of $50,000 per QALY, naltrexone plus counselling would be considered a cost-effective treatment for detoxified patients.

The study by Walters et al. (2009) evaluated the cost-effectiveness of cognitive behavioural therapy plus naltrexone (CBT + NTX) vs CBT alone and did not specify the perspective chosen but most likely used the perspective of the health sector since costs included personnel, contracted services, supplies, equipment and facilities. Walters et al. (2009) found an increase in utilities from pre- to post-treatment but no significant difference between groups. CBT + NTX was more costly than CBT alone, but patients achieved a higher rate of session completion and remaining abstinent (CBT + NTX 62.6% vs CBT alone 36.1%), leading to the conclusion of weak dominance for CBT + NTX.

The previously mentioned Dunlap et al. (2010) and Zarkin et al. (2008) also evaluated medical management MM + NTX and MM + NTX + CBI. Dunlap et al. (2010) found that from a patient perspective, MM + NTX vs MM + placebo would result in $1904 per patient avoiding a return to heavy drinking or $28 per patient for a 1% increase in days abstinent. MM + NTX + CBI was more costly and less effective leading to it being dominated. Meanwhile, the result from Zarkin et al. (2008), utilising the perspective of a treatment provider, found an ICER of $5300/patient avoiding heavy drinking for the comparison of MM + NTX vs MM + placebo. Zarkin et al. (2008) also found that when CBI is added to MM + NTX this becomes economically dominated.

#### Naltrexone and acamprosate

Dunlap et al. (2010) and Zarkin et al. (2008) also evaluated medical management plus a combination of naltrexone and acamprosate (MM + NTX + ACP). These two studies evaluated three different outcome measures: percent days abstinent, the proportion of patients with good clinical outcomes, and the proportion of patients that do not return to heavy drinking. Overall, both studies found that MM + NTX + ACP was slightly more costly and slightly more effective than MM + NTX across the three different outcome measures. When CBI was added, the intervention (MM + NTX + ACP + CBI) became dominant, more costly and less effective. Sensitivity analyses from both studies showed inconsistencies in the results when different scenarios were analysed. Both studies stated that results were sensitive to the price of acamprosate (Dunlap et al., 2010) or naltrexone (Zarkin et al., 2008). In addition, according to Dunlap et al. (2010), MM + NTX + ACP had the highest probability of being cost-effective if generic drugs were used, but if patients lacked insurance coverage MM + placebo became the cost-effective option.

A cost-utility analysis in the Netherlands by Sluiter et al. (2018) compared genotype-guided treatments of acamprosate or naltrexone and standard care (random allocation to acamprosate or naltrexone). From a societal perspective, genotype-guided therapy versus standard care would produce an ICER of $27,127 per QALY gained. However, genotype-guided therapy has not been validated by other efficacy studies.

Another CUA study by Avanceña et al. (2021) used an economic model to evaluate FDA-approved medications (acamprosate and naltrexone), commonly used but not FDA-approved medications (baclofen, gabapentin and topiramate), counselling alone and ‘do-nothing’ to treat alcohol use disorder among patients with compensated alcohol-related cirrhosis. All medications were dominant compared to the do-nothing alternative from both societal and healthcare perspectives (lower costs and more QALYs gained). When the ICER was compared to the next most expensive, undominated option, acamprosate and naltrexone were dominant over baclofen, gabapentin and topiramate, counselling and ‘do-nothing’.

#### Reporting quality standards assessment

Quality assessment items on the CHEC-list that were satisfied by all 15 studies were: a well-defined research question, a full economic evaluation with the comparison of costs and outcomes of two or more alternatives, and reported adequate conclusions justified by the reported data (Items 3, 4, 16). Of the 15 studies included, 11 (73%) studies have fully identified all relevant costs in relation to the perspective chosen (Item 7), while only 9 (60%) studies have indicated any information relating to conflict of interests between researchers and funders (Item 18). The most common drawbacks were found in 11 (73%) studies that inadequately reported ethical aspects and distributional implications (Item 19) and in 8 (53%) studies that vaguely described competing alternatives omitting relevant factors such as intensity, duration and frequency of the interventions (Item 2). Overall, the quality scores of the included studies range from 63% to 95%, of which the majority of the studies (*n* = 8) have a score above 80%. A summary of the quality assessment is shown in Table 2.

**Table 2.** Quality assessment using CHEC list.

	Annemans et al. (2000)	Avancena et al. (2020)	Brodtkorb et al. (2016)	Cobiac et al. (2009)	Dunlap et al. (2010)	Laramee et al. (2014)	Laramee et al. (2016)	Millier et al. (2017)	Mortimer and Segal (2005)	Palmer et al. (2000)	Rychlik et al. (2003)	Schädlich and Brecht (1998)	Sluiter et al. (2018)	Walters et al. (2009)	Zarkin et al. (2008)
1. Is the study population clearly described?	No	Yes	No	Yes	No	Yes	Yes	Yes	Yes	Yes	Yes	Yes	No	Yes	Yes
2. Are competing alternatives clearly described?	Yes	No	No	Yes	Yes	No	No	No	Yes	Yes	Yes	No	No	Yes	No
3. Is a well-defined research question posed in answerable form?	Yes	Yes	Yes	Yes	Yes	Yes	Yes	Yes	Yes	Yes	Yes	Yes	Yes	Yes	Yes
4. Is the economic study design appropriate to the stated objective?	Yes	Yes	Yes	Yes	Yes	Yes	Yes	Yes	Yes	Yes	Yes	Yes	Yes	Yes	Yes
5. Is the chosen time horizon appropriate to include relevant costs and consequences?	Yes	Yes	Yes	Yes	No	Yes	Yes	Yes	Yes	Yes	Yes	Yes	Yes	No	No
6. Is the actual perspective chosen appropriate?	Yes	Yes	Yes	Yes	Yes	Yes	Yes	Yes	Yes	Yes	No	Yes	Yes	No	Yes
7. Are all important and relevant costs for each alternative identified?	Yes	Yes	Yes	Yes	Yes	Yes	Yes	Yes	No	Yes	No	Yes	No	No	Yes
8. Are all costs measured appropriately in physical units?	Yes	Yes	Yes	Yes	Yes	Yes	Yes	Yes	No	Yes	Yes	Yes	No	Yes	Yes
9. Are costs valued appropriately?	Yes	Yes	Yes	Yes	Yes	Yes	Yes	Yes	No	Yes	Yes	Yes	Yes	No	Yes
10. Are all important and relevant outcomes for each alternative identified?	Yes	Yes	Yes	Yes	Yes	Yes	Yes	Yes	Yes	Yes	Yes	Yes	No	Yes	Yes
11. Are all outcomes measured appropriately?	Yes	Yes	No	Yes	Yes	Yes	Yes	Yes	Yes	Yes	Yes	Yes	Yes	Yes	Yes
12. Are outcomes valued appropriately?	No	Yes	No	Yes	Yes	Yes	Yes	Yes	Yes	Yes	Yes	Yes	Yes	Yes	Yes
13. Is an incremental analysis of costs and outcomes of alternatives performed?	Yes	Yes	Yes	Yes	Yes	Yes	Yes	Yes	Yes	Yes	No	Yes	Yes	No	Yes
14. Are all future costs and outcomes discounted appropriately?	No	Yes	Yes	Yes	Yes	Yes	Yes	Yes	Yes	Yes	Yes	Yes	Yes	Yes	Yes
15. Are all important variables, whose values are uncertain, appropriately subjected to sensitivity analysis?	Yes	Yes	Yes	Yes	Yes	Yes	Yes	Yes	Yes	Yes	No	Yes	Yes	No	Yes
16. Do the conclusions follow from the data reported?	Yes	Yes	Yes	Yes	Yes	Yes	Yes	Yes	Yes	Yes	Yes	Yes	Yes	Yes	Yes
17. Does the study discuss the Generalisability of the results to other settings and patient/client groups?	Yes	Yes	Yes	Yes	Yes	Yes	Yes	No	Yes	Yes	Yes	Yes	Yes	Yes	Yes
18. Does the article indicate that there is no potential conflict of interest between study researcher(s) and funder(s)?	No	Yes	Yes	Yes	No	Yes	Yes	No	Yes	No	No	No	Yes	Yes	Yes
19. Are ethical and distributional issues discussed appropriately?	No	Yes	No	No	Yes	Yes	Yes	No	No	No	No	No	No	No	No
Yes	14	18	14	18	16	18	18	15	15	17	13	16	13	12	16
No	5	1	5	1	3	1	1	4	4	2	6	3	6	7	3
Quality score	74%	95%	74%	95%	84%	95%	95%	79%	79%	89%	68%	84%	68%	63%	84%

‘Yes’ indicates that the study adequately addressed specific criteria; ‘No’ indicates that the criteria have not been fulfilled or inadequate information is available in the published material.

#### Colour coded cost-effectiveness results

**Figure 2.**
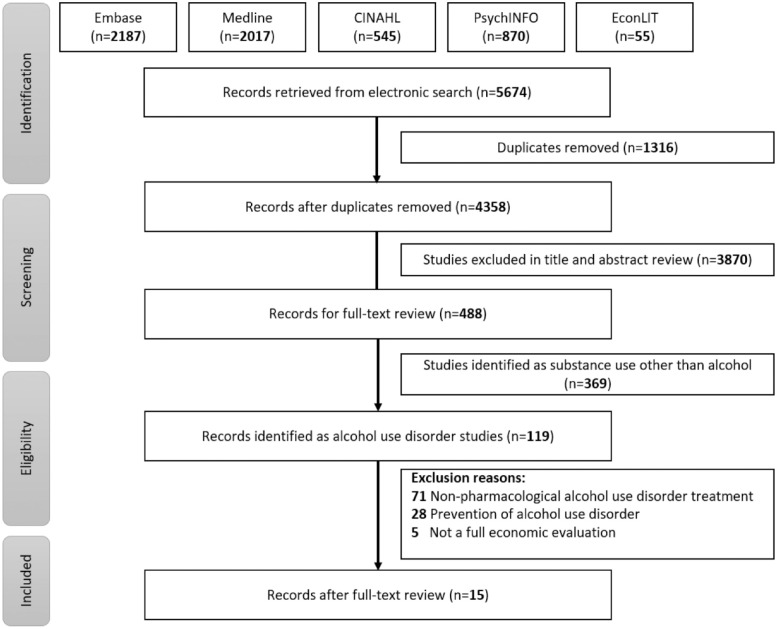
Colour-coded cost-effectiveness.

Figure 2 presents a summary of different interventions grouped according to drug names and their results graded based on a decision-making perspective as either likely to be favoured, rejected or unclear. All five evaluations of nalmefene found it to be cost-effective in treating adults with alcohol use disorder and were graded as ‘favourable’. Acamprosate was also evaluated in six analyses, four found cost-savings and two were categorised as ‘reject’ for being dominated by other interventions. Among five of the naltrexone interventions, one was considered cost-effective, one categorised as ‘reject’, while three were categorised as ‘unclear’ since they produced improved outcomes at a higher cost. Two studies evaluated the combination of naltrexone and acamprosate, and both were also graded as ‘unclear’. Three interventions including either naltrexone or acamprosate were considered ‘favourable’ or cost-effective.

Overall, 62% of the pharmacological interventions were ‘favourable’, 24% were categorised as ‘unclear’, and 14% of the interventions were categorised as ‘rejected’.

## Discussion

Based on the evidence presented in this systematic review, pharmacotherapies for alcohol use disorders can be considered cost-effective in addition to psychosocial support or medical management. Two studies from societal perspectives found that the addition of pharmacotherapy to psychosocial support was a dominant strategy meaning that it was cost-saving and led to improved outcomes (Brodtkorb et al., 2016; Millier et al., 2017). An additional societal perspective analysis found that pharmacotherapy (naltrexone + counselling) was more costly and more effective than counselling alone, with an incremental cost/QALY ratio falling below the accepted WTP threshold (Mortimer and Segal, 2005). From more limited healthcare payer perspectives, five studies found that pharmacotherapy added to psychosocial support was dominant (less costly and more effective) or cost-effective, accruing additional benefits at a higher cost but under accepted willingness to pay thresholds (Avanceña et al., 2021; Laramee et al., 2014, 2016; Millier et al., 2017; Walters et al., 2009). These results emphasise that strategies to reduce the economic burden of heavy and chronic alcohol consumption should consider the widespread implementation of pharmacotherapy for the management of AUD.

Several methodological issues exist in economic evaluations which can lead to difficulty in comparing economic evaluations of treatments for AUDs and can influence the applicability of economic evaluations and their interpretation. Heterogeneity in a range of methodological factors occurs across the studies and between countries including outcome measures, costing methodology, perspective, healthcare system delivery and reimbursement and these can compromise comparisons of cost data across studies (Drummond et al., 2009). For example, some economic evaluation methods measure outcomes in different units. Cost-effectiveness analyses use ‘natural units’ for outcomes assessment such as percentage days abstinent or percentage reduction in total consumption. These outcomes are typically unproblematic to collect and may be useful for clinical decision-making. However, it is important to address incomparability of results, varied value assignments, heterogeneity in the study population and confounding factors when comparing CEA studies that use different outcome measures as these differences can introduce biased conclusions about the overall cost-effectiveness of an intervention. Several of the economic evaluations in this review reported cost-effectiveness ratios (i.e. cost per abstinent patient, cost per good clinical outcome) (Dunlap et al., 2010; Schädlich and Brecht, 1998). When the intervention is dominant the interpretation is straightforward, as seen in two studies evaluating the addition of nalmefene to standard psychosocial therapy. Nonetheless, it is difficult to interpret the value for money represented by these results in that there are additional benefits at an additional cost, but these benefits are not described in natural units. For example, the studies evaluating naltrexone or the combination of naltrexone and acamprosate added to standard medical management found that naltrexone and the combination of naltrexone and acamprosate were more costly while providing additional benefits measured as good clinical outcomes, avoiding a return to heavy drinking, and days abstinent for additional cost (Dunlap et al., 2010; Zarkin et al., 2008).

One approach to this challenge is to consider cost-utility analyses using cost per QALYs gained or DALYs averted which has the benefit of an outcome measure that can be compared across different therapeutic areas. Cost-utility analyses also have inherent value-for-money connotations since cost/QALY thresholds are commonly used to make reimbursement decisions by health technology assessment agencies such as the UK’s NICE and Australia’s Pharmaceutical Benefits Advisory Committee. Five cost-utility analyses suggest that nalmefene or naltrexone plus psychosocial support/counselling/CBT would be cost-effective relative to these supports alone (Brodtkorb et al., 2016; Laramee et al., 2014, 2016; Millier et al., 2017; Mortimer and Segal, 2005; Walters et al., 2009). Additional cost-utility analysis modelling estimated that acamprosate and naltrexone would be cost-saving compared to off-label medications, counselling or no treatment (Avanceña et al., 2021). Clearly, the choice of a comparator is a key factor in determining the outcome and conclusions from economic evaluations. For example, the comparison of naltrexone in residential treatment compared to seven other policies to reduce alcohol harm (i.e. volumetric taxation, advertising bans, random breath testing, etc) was a comparison of prevention policies with treatment more broadly, wherein naltrexone plus residential treatment represented treatment, where the resulting ICER was well above the WTP threshold (Cobiac et al., 2009).

Finally, economic evaluations can be incorporated into a clinical trial or estimated from an economic model. Trial-based economic evaluations typically have limited time horizons, numbers of study sites and participants, but benefit from greater precision in the collection of cost and outcome data. Economic models allow costs and outcomes to be estimated over longer time horizons and for multiple comparators but are only as good as the underlying data utilised in their construction. This is highlighted by the nalmefene studies included in this review that relied on economic models (Laramee et al., 2014, 2016; Millier et al., 2017). All three utilised effectiveness data derived from three clinical trials sponsored by the manufacturer that showed nalmefene to be effective over a six-month to one-year time frame. Subsequent meta-analyses reported mixed results regarding effectiveness (Mann et al., 2016; Palpacuer et al., 2015), leading to uncertainty surrounding the validity of the economic analyses (Naudet et al., 2016a, 2016b).

### Limitations and recommendations

This review is subject to limitations. It only included peer-reviewed, English language publications, missing return on investment studies published in reports rather than academic journals. The search criteria also excluded economic evaluations that involved participants with substance use disorders other than alcohol. We were also unable to undertake a meta-analysis due to study heterogeneity and the limited number of economic evaluations available. Finally, the use of the CHEC-list to assess study quality is a limitation since it is designed for trial-based economic evaluations and no weighting exists for each criterion. Although the criteria listed in the CHEC-list provide valuable information about the quality of economic evaluation studies, some of them tend to be subjective, posing a challenge to inter-rater reliability (Evers et al., 2005). Nonetheless, the current review highlights that more economic evaluations of the treatment of alcohol use disorders are required. Incorporating appropriate data collection into clinical trials will allow trial-based economic evaluations to be undertaken and provide data for model-based economic evaluations to support future decision-making.

## Conclusion

The few available full economic evaluations presented in this review generally indicate that the utilisation of pharmacotherapies in addition to psychosocial support or medical management for the treatment of alcohol use disorder would be cost-effective. This is especially evident when the societal costs of alcohol consumption are considered. While this suggests an increased role for alcohol pharmacotherapy in strategies to expand access to treatment and enhance uptake to reduce the burden of alcohol, additional economic evaluations of these therapies specific to Australia would provide useful information to improve the prescribing of these medications.

## Supplemental Material

sj-docx-1-anp-10.1177_00048674231201541 – Supplemental material for Economic evaluations of alcohol pharmacotherapy: Systematic review of economic evaluations of pharmacotherapy for the treatment of alcohol use disorder Click here for additional data file. Supplemental material, sj-docx-1-anp-10.1177_00048674231201541 for Economic evaluations of alcohol pharmacotherapy: Systematic review of economic evaluations of pharmacotherapy for the treatment of alcohol use disorder by Benjamin Higginbotham, Joahna Kevin Perez, Eva Louie, Paul S Haber, Dan Lubman, Shalini Arunogiri, Mary Lou Chatterton and Kirsten C Morley in Australian & New Zealand Journal of Psychiatry
