# Supplementary material for: Economic evaluations of alcohol pharmacotherapy: Systematic review of economic evaluations of pharmacotherapy for the treatment of alcohol use disorder
Source: Aust N Z J Psychiatry. 2023 Oct 12;58(2):117–33. doi: 10.1177/00048674231201541 (PMC10838482; doi:10.1177/00048674231201541)
Supplement: sj-docx-1-anp-10.1177_00048674231201541 – Supplemental material for Economic evaluations of alcohol pharmacotherapy: Systematic review of economic evaluations of pharmacotherapy for the treatment of alcohol use disorder [file sj-docx-1-anp-10.1177_00048674231201541.docx]

Systematic review of economic evaluations of pharmacotherapy for the treatment of alcohol use disorder

Supplementary Table 1: Search strategy (Embase)

| No. | Query | Results  (29/09/2022) |
| --- | --- | --- |
| #1 | 'alcohol abuse'/exp | 45,919 |
| #2 | (alcohol NEAR/2 (use OR misuse OR abuse OR intake OR consum*)):ti | 36,004 |
| #3 | (alcohol NEAR/2 (use OR misuse OR abuse OR intake OR consum*)):ab | 163,966 |
| #4 | #1 OR #2 OR #3 | 190,473 |
| #5 | treat*:ti OR manag*:ti | 2,684,653 |
| #6 | treat*:ab OR manag*:ab | 3,826,051 |
| #7 | #5 OR #6 | 4,129,802 |
| #8 | pharma*:ti | 412,199 |
| #9 | Pharma*:ab | 1,123,423 |
| #10 | #8 OR #9 | 1,300,235 |
| #11 | 'cost benefit analysis'/de | 91,309 |
| #12 | 'cost benefit analysis':ti | 1,938 |
| #13 | 'cost benefit analysis':ab | 4,352 |
| #14 | 'economic evaluation'/exp | 338,296 |
| #15 | 'economic evaluation*':ti | 8,067 |
| #16 | 'economic evaluation*':ab | 15,459 |
| #17 | 'cost analys?s':ti | 4,847 |
| #18 | 'cost analys?s':ab | 9,735 |
| #19 | 'return on investment':ti | 525 |
| #20 | 'return on investment':ab | 2,670 |
| #21 | 'return to investment':ti | 1 |
| #22 | 'return to investment':ab | 9 |
| #23 | 'cost effectiveness analysis'/de | 170,828 |
| #24 | 'cost effectiveness analysis':ti | 9,018 |
| #25 | 'cost effectiveness analysis':ab | 11,108 |
| #26 | 'cost utility analysis'/de | 11,307 |
| #27 | 'cost utility analysis':ti | 1,986 |
| #28 | 'cost utility analysis':ab | 3,267 |
| #29 | #11 OR #12 OR #13 OR #14 OR #15 OR #16 OR #17 OR #18 OR #19 OR #20 OR #21 OR #22 OR #23 OR #24 OR #25 OR #26 OR #27 OR #28 | 347,583 |
| #30 | #7 OR #10 | 5,215,811 |
| #31 | #4 AND #29 AND #30 | 395 |
| #32 | #4 AND #29 AND #30 AND [humans]/lim AND [english]/lim AND [embase]/lim | 332 |

Search strategy (Medline)

| # | Query | Results  (29/09/2022) |
| --- | --- | --- |
| S1 | (MH "Alcoholism") OR (MH "Binge Drinking") OR (MH "Alcoholic Intoxication") | 91,527 |
| S2 | (MH "Alcohol Drinking+") | 76,180 |
| S3 | TI (alcohol) N2 (use or misuse or abuse or intake or consum*) | 30,130 |
| S4 | AB (alcohol) N2 (use or misuse or abuse or intake or consum*) | 121,600 |
| S5 | S1 OR S2 OR S3 OR S4 | 216,069 |
| S6 | TI treat* or manag* | 3,499,428 |
| S7 | AB treat* or manag* | 7,049,398 |
| S8 | S6 OR S7 | 7,681,891 |
| S9 | TI pharma* | 276,233 |
| S10 | AB pharma* | 770,884 |
| S11 | S9 OR S10 | 883,776 |
| S12 | (MH "Cost-Benefit Analysis") | 90,527 |
| S13 | TI "Cost Benefit Analys?s" | 1,513 |
| S14 | AB "Cost Benefit Analys?s" | 3,980 |
| S15 | TI "economic evaluation*" | 5,906 |
| S16 | AB "economic evaluation*" | 10,980 |
| S17 | (MH "Costs and Cost Analysis") | 50,811 |
| S18 | TI "Cost Analys?s" | 3,217 |
| S19 | AB "Cost Analys?s" | 6,088 |
| S20 | TI "return on investment" | 442 |
| S21 | AB "return on investment" | 2,007 |
| S22 | TI "return to investment" | 1 |
| S23 | AB "return to investment" | 5 |
| S24 | TI "cost effectiveness analys?s" | 6,138 |
| S25 | AB "cost effectiveness analys?s" | 9,795 |
| S26 | TI "cost utility analys?s" | 1,297 |
| S27 | AB "cost utility analys?s" | 2,893 |
| S28 | S12 OR S13 OR S14 OR S15 OR S16 OR S17 OR S18 OR S19 OR S20 OR S21 OR S22 OR S23 OR S24 OR S25 OR S26 OR S27 | 155,319 |
| S29 | S8 AND S11 | 8,180,564 |
| S30 | S5 AND S28 AND S29 (Limiters - English Language; Human; Publication Type: Introductory Journal Article, Journal Article Search modes - Boolean/Phrase) | 470 |

Search strategy (Cinahl)

| # | Query | Results  (29/09/2022) |
| --- | --- | --- |
| S1 | (MH "Alcoholism") OR (MH "Alcohol Drinking+") | 50,285 |
| S2 | TI (alcohol) N2 (use or misuse or abuse or intake or consum*) | 16,147 |
| S3 | AB (alcohol) N2 (use or misuse or abuse or intake or consum*) | 48,192 |
| S4 | S1 OR S2 OR S3 | 78,739 |
| S5 | TI treat* OR manag* | 1,058,771 |
| S6 | AB treat* OR manag* | 1,622,050 |
| S7 | S5 OR S6 | 1,782,783 |
| S8 | TI pharma* | 64,388 |
| S9 | AB pharma* | 134,048 |
| S10 | S8 OR S9 | 165,870 |
| S11 | (MH "Cost Benefit Analysis") | 38,291 |
| S12 | TI "Cost Benefit Analys?s" | 419 |
| S13 | AB "Cost Benefit Analys?s" | 1,040 |
| S14 | TI "economic evaluation*" | 2,831 |
| S15 | AB "economic evaluation*" | 4,554 |
| S16 | (MH "Costs and Cost Analysis") | 19,271 |
| S17 | TI "Cost Analys?s" | 1,324 |
| S18 | AB "Cost Analys?s" | 1,890 |
| S19 | TI "return on investment" | 346 |
| S20 | AB "return on investment" | 1,096 |
| S21 | TI "return to investment" | 3,350 |
| S22 | AB "return to investment" | 1 |
| S23 | TI "cost effectiveness analys?s" | 3,003 |
| S24 | AB "cost effectiveness analys?s" | 3,688 |
| S25 | TI "cost utility analys?s" | 662 |
| S26 | AB "cost utility analys?s" | 1,089 |
| S27 | S11 OR S12 OR S13 OR S14 OR S15 OR S16 OR S17 OR S18 OR S19 OR S20 OR S21 OR S22 OR S23 OR S24 OR S25 OR S26 | 63,098 |
| S28 | S7 OR S10 | 1,864,857 |
| S29 | S4 AND S27 AND S28 (Limiters - English Language; Peer Reviewed; Human; Publication Type: Corrected Article, Journal Article) | 111 |

Search strategy (PsychInfo)

| # | Query | Results  (29/09/2022) |
| --- | --- | --- |
| S1 | TI (alcohol) N2 (use or misuse or abuse or intake or consum*) | 20,266 |
| S2 | DE "Alcohol Abuse" OR DE "Alcoholism" OR DE "Binge Drinking" OR DE "Alcohol Drinking Attitudes" OR DE "Alcohol Drinking Patterns" OR DE "Social Drinking" OR DE "Underage Drinking" OR DE "Alcohol Use Disorder" OR DE "Alcohol Abuse" OR DE "Alcohol Intoxication" | 81,919 |
| S3 | AB (alcohol) N2 (use or misuse or abuse or intake or consum*) | 70,401 |
| S4 | DE “Alcohol Use Disorder” | 3,293 |
| S5 | S1 OR S2 OR S3 OR S4 | 111,296 |
| S6 | TI treat* OR manag* | 651,237 |
| S7 | AB treat* OR manag* | 1,164,311 |
| S8 | S6 OR S7 | 1,187,402 |
| S9 | TI pharma* | 18,550 |
| S10 | AB pharma* | 87,099 |
| S11 | S9 AND S10 | 92,131 |
| S12 | TI "Cost Benefit Analys?s" | 208 |
| S13 | AB "Cost Benefit Analys?s" | 1,108 |
| S14 | TI "economic evaluation*" | 684 |
| S15 | AB "economic evaluation*" | 1,785 |
| S16 | DE "Costs and Cost Analysis" | 19,830 |
| S17 | TI "Cost Analys?s" | 222 |
| S18 | AB "Cost Analys?s" | 719 |
| S19 | TI "return on investment" | 141 |
| S20 | AB "return on investment" | 949 |
| S21 | TI "return to investment" | 141 |
| S22 | AB "return to investment" | 949 |
| S23 | TI "cost effectiveness analys?s" | 459 |
| S24 | AB "cost effectiveness analys?s" | 1,250 |
| S25 | TI "cost utility analys?s" | 92 |
| S26 | AB "cost utility analys?s" | 398 |
| S27 | S12 OR S13 OR S14 OR S15 OR S16 OR S17 OR S18 OR S19 OR S20 OR S21 OR S22 OR S23 OR S24 OR S25 OR S26 | 23,576 |
| S28 | S8 OR S11 | 1,223,320 |
| S29 | S5 AND S27 AND S28 (Limiters - Peer Reviewed; Publication Type: Peer Reviewed Journal; English; Population Group: Human; Document Type: Erratum/Correction, Journal Article, Retraction) | 224 |

Search strategy (EconLit)

| # | Query | Results  (29/09/2022) |
| --- | --- | --- |
| S1 | (ZW "alcohol") or (ZW "alcohol availability, health, alcohol consumption") or (ZW "alcohol consumption") or (ZW "alcohol consumption, alcohol tax, binge drinking, beer, wine and spirits") or (ZW "alcohol misuse, morbidity, ordered probit, tobit, eq5d") or (ZW "alcohol, demand model, patterns of consumption") or (ZW "alcohol.") or (ZW "alcoholism") | 58 |
| S2 | TI (alcohol) N2 (use or misuse or abuse or intake or consum*) | 348 |
| S3 | AB (alcohol) N2 (use or misuse or abuse or intake or consum*) | 962 |
| S4 | S1 OR S2 OR S3 | 1,116 |
| S5 | TI treat* or manag* | 329,955 |
| S6 | AB treat* or manag* | 355,074 |
| S7 | S5 OR S6 | 357,516 |
| S8 | TI pharma* | 2,679 |
| S9 | AB pharma* | 3,868 |
| S10 | S8 OR S9 | 5,076 |
| S11 | (ZW "cost benefit") or (ZW "cost benefit analysis") | 1,114 |
| S12 | TI "Cost Benefit Analys?s" | 1,041 |
| S13 | AB "Cost Benefit Analys?s" | 2,024 |
| S14 | (ZW "economic evaluation") | 2 |
| S15 | TI "economic evaluation*" | 862 |
| S16 | AB "economic evaluation*" | 929 |
| S17 | (ZW "cost analysis") | 1 |
| S18 | TI "cost analys?s" | 660 |
| S19 | AB "cost analys?s" | 897 |
| S20 | (ZW "return on investment") | 11 |
| S21 | TI "return on investment" | 137 |
| S22 | AB "return on investment" | 750 |
| S23 | TI "return to investment" | 137 |
| S24 | AB "return to investment" | 750 |
| S25 | (ZW "cost effective") or (ZW "cost effectiveness") or (ZW "cost effectiveness analysis") | 736 |
| S26 | TI "cost effectiveness analys?s" | 350 |
| S27 | AB "cost effectiveness analys?s" | 486 |
| S28 | TI "cost utility analys?s" | 64 |
| S29 | AB "cost utility analys?s" | 129 |
| S30 | S11 OR S12 OR S13 OR S14 OR S15 OR S16 OR S17 OR S18 OR S19 OR S20 OR S21 OR S22 OR S23 OR S24 OR S25 OR S26 OR S27 OR S28 OR S29 | 7,517 |
| S31 | S7 OR S10 | 360,652 |
| S32 | S4 AND S30 AND 31 (Limiters - Publication Type: Journal Article Search modes - Boolean/Phrase) | 2 |

**Supplementary Table 2: Summary of sensitivity analyses of included studies**

| **Lead author (year), country** | **Interventions and Comparator** | **Perspective,  Time horizon** | **Currency, Year of pricing, Discount rates** | **Sensitivity Analyses, WTP** |
| --- | --- | --- | --- | --- |
| **Nalmefene** |  |  |  |  |
| Brodtkorb et al. (2016), UK | (1) Nalmefene + psychosocial support (BRENDA)  (2) Psychosocial support (BRENDA) | Societal, 5 years | UK Pound (£); Ref year: 2012; Discount rate: 3.5% | PSA and OWSA: Nalmefene + pyschosocial support were dominant (Q2);  Scenario Analysis:  - 1-year time horizon: incremental cost: -£1,011 (AUD -2,503)  incremental QALYs: 0.017  - Societal cost of criminal harm (no productivity cost):  incremental cost: -£2,483 (AUD -6,147)  incremental QALYs: 0.086  WTP: £20,000 (AUD 49,511)/QALY gained |
| Millier et al. (2017), UK | (1) Nalmefene + psychosocial support  (2) Psychosocial support only (3) No treatment | Societal perspective and Third-party payer (TPP), 1 and 5 years | UK Pound (£); Ref year: 2011; Discount rate: 3.5% | One-way DSA:  NMF + PS vs PS only: -TPP perspective (5 years) dominant with all ICERs below WTP.  -Societal perspective (all years) dominant  NMF + PS vs No treatment: -Societal perspective (all years) dominant  PSA:  NMF + PS vs PS only:  -TPP perspective 42% (1year) and 56% (5 years) probability of being cost-effective at £20,000/QALY WTP  -Societal perspective around 70% probability of being cost-effective at £20,000/QALY WTP for all years  WTP: £20,000 (AUD 50,285)/QALY gained |
| Laramee et al. (2014),  UK | (1) Nalmefene + psychosocial support (BRENDA) (2) psychosocial support only (BRENDA) | National Health Service (NHS) in England and Wales, 5 years | UK Pound (£); Ref year: 2010/11; Discount rate: 3.5% | PSA: -94% probability of being cost-effective at £20,000/QALY WTP  OWSA:  -NMF + psychosocial is still cost-effective in all OWSAs  WTP: £20,000 (AUD 51,250)/ QALY gained |
| Laramee et al. (2016), UK | (1) Nalmefene + psychosocial support (BRENDA) (2) psychosocial support only (BRENDA) | National Health Service (NHS), 5 years | UK Pound (£); Ref year: 2012; Discount rate: 3.5% | PSA:  -uncertainty around parameters did not alter base-case result- dominant  Scenario Analysis:  -changes in patient dropping out would enter high or very high DRL states: dominant  WTP: £20,000 (AUD 49,511)/QALY gained |
| **Acamprosate** |  |  |  |  |
| Rychlik et al. (2003), Germany | (1) Adjuvant acamprosate therapy (2) Standard psychosocial rehabilitation program, no pharmacotherapy (standard cohort) | not clearly specified, 1 year | EUR;  Ref year: not clearly specified (assuming 1998/99 when the study was performed) Discount rate: 0 | No sensitivity analysis was reported |
| Schadlich et al. (1998), Germany | (1) Adjuvant acamprosate therapy (2) Standard psychosocial rehabilitation program, no pharmacotherapy (standard cohort) | German healthcare system & Statutory Health Insurance scheme, 10 years | German Marks (DM); Ref year: 1995 Discount rate: 5% | -base case result of cost-saving remained robust in the SA.  -Impact of uncertainties of model parameters on the target variable is much lower than the impact of effect parameter. |
| Palmer et al. (2000), Germany | (1) standard counselling therapy + 48 weeks of adjuvant Acamprosate  (2) standard counselling therapy only | German health insurance perspective, Lifetime | German Marks (DM); Ref year: 1996 Discount rate: 5% | OWSA:  -assumed both groups have equal abstinence rate:  = 0.59 life years gained (non-discounted)  =cost-savings DEM 460 (AUD 603) (discounted)  -assumed no effect of abstinence on suicide rate:  =0.75 life years gained (non-discounted)  =cost-savings DEM 2,024 (AUD 2,652) (discounted)  -if 48weeks acquisition costs of acamprosate were below DEM 4,000 (AUD 5,240) then intervention lead to cost-savings over a patient’s lifetime. |
| Annemans et al. (2000),  Belgium | (1) Acamprosate (Campral)  (2) Placebo | Belgian health insurance perspective,  2 years | Belgian Francs (BEF);  Ref year: not stated  Discount rate: not stated | SA:  -if 24% stayed for follow-up in an institution acamprosate is still cost saving.  -varying cost of acute hospitalisation (i.e. cost reduced by more than 50%) then acamprosate would not be cost saving |
| **Naltrexone** |  |  |  |  |
| Cobiac et al. (2009), Australia | (1) volumetric taxation (2)advertising bans (3)increase in minimum legal drinking age (4)licensing controls on operating hours (5)brief intervention (with and without GP telemarketing and support) (6)drink driving campaigns (7)random breath testing (8)residential treatment for alcohol dependence (with and without Naltrexone); each intervention is compared to current practice | Australian Health care system, Lifetime | AUD ($); Ref year: 2003; Discount rate: 3% | Residential treatment with naltrexone SA:  -undiscounted: not-cost saving; Median ICER $71,000 (AUD 115,283); 2% probability of being cost-effective under $50,000/DALY    -varying decay rates (0-100%) of intervention effects still not cost-effective  WTP:  $50,000 (AUD 81,185) /DALY gained |
| Mortimer et al. (2005), Australia | (1) Brief interventions for problem drinkers vs No alcohol-related treatment  (2) Psychotherapy for mild to moderate dependence vs Behavioural self-control training (BSCT)  (3) Drug-therapy (naltrexone) + counselling for detoxified patients with a history of severe physical dependence vs Placebo + counselling | Societal perspective, Lifetime | AUD ($); Ref year: 2003; Discount rate: 5% | Naltrexone + counselling vs Placebo + counselling  Univariate SA:  -if Naltrexone + counselling increased in initial relapse rate by a factor of three = placebo arm is dominant.  -$/QALY range from SA = $3,725 (AUD 6,048) to infinite  Threshold SA:  -$/year for Naltrexone+ counselling to dominate = -$3,008/year (AUD -4884)  WTP:  $50,000 (AUD 81,185) /QALY gained |
| Walters et al. (2009) (Australia) | (1) CBT + Naltrexone (2) CBT alone | not clearly specified, most likely treatment provider perspective, 12 weeks | AUD ($); Ref year: not stated Discount rate: 0 | No sensitivity analysis was reported |
| **Naltrexone and/or acamprosate** |  |  |  |  |
| Dunlap et al. (2010), USA | (1)Medical Management (MM) + placebo (2)MM + Naltrexone (3)MM + Naltrexone +Acamprosate (4)Combined behavioural intervention (CBI) only (5)MM + Acamprosate  (6)MM+placebo + CBI (7)MM + Naltrexone + CBI (8)MM + Naltrexone +Acamprosate + CBI (9) MM + Acamprosate +CBI | Patient perspective,  16 weeks | US Dollars ($); Ref year: 2007 Discount rates: 0 | SA: scenario 1: generic drug options (same copay for both drugs) -yielded slightly lower costs for treatments that include Acamprosate -(3)MM + Nalterxone + Acamprosate has the highest probability of being the most cost-effective except at small WTP values (<$500) (AUD <930)  scenario 2: no insurance coverage -yields greater costs for patients compared to base case -(1) MM+ placebo has the highest probability of being most cost-effective at much greater WTP values (<$1,000) (AUD <1,862 )  * both scenarios assumed no changes in treatment effectiveness |
| Zarkin et al. (2008), USA | (1)Medical Management (MM) + placebo (2)MM + Naltrexone (3)MM + Naltrexone +Acamprosate (4)Combined behavioural intervention (CBI) only (5)MM + Acamprosate  (6)MM+placebo + CBI (7)MM + Naltrexone + CBI (8)MM + Naltrexone +Acamprosate + CBI (9) MM + Acamprosate +CBI | Treatment provider, 16 weeks | USD ($); Ref year: 2007 Discount rate: 0 | OWSA:  -Using alternative pharmaceutical prices:  Percent days abstinent  MM + placebo = cost-effective  MM + placebo + CBI = cost-effective  MM + Naltrexone + Acamprosate = cost-effective  MM + Naltrexone = not cost-effective  Proportion of patients who avoid heavy drinking  MM + placebo = cost-effective  MM + Acamprosate = cost-effective  MM + Naltrexone + Acamprosate = cost-effective  MM + Naltrexone = not cost-effective  Proportion of patients with good clinical outcome: MM + placebo = cost-effective  MM + placebo + CBI = cost-effective  MM + Naltrexone + Acamprosate = cost-effective  MM + Naltrexone = not cost-effective  -Varying labour costs (staff wages):  Results are not sensitive to changes in wages  TWSA:  -varied both pharmaceutical prices and staff wages:  Results are the same as OWSA |
| Sluiter et al. (2018), Netherlands | (1) Genotype-guided treatment (G-allele carriers receiving naltrexone; AA homozygotes acamprosate or naltrexone)  (2) Standard care (random treatment allocation to acamprosate or naltrexone) | Societal perspective, 1 year | EUR;  Ref year: 2015 Discount rate: 0 | OWSA (variation per parameters within an uncertainty range):  -risk ratio to relapse in screening with naltrexone ranging from not cost-effective at EUR -105 (AUD -213) to cost-effective at EUR 684 (AUD 1,390).  -all other parameters iNMB stays positive.  Threshold analysis (with changes in risk ratios):  -Risk ratio <0.81 screening with naltrexone is cost-effective at EUR 80,000/QALY WTP  -Risk ratio <0.25 screening with naltrexone becomes dominant  WTP: -EUR 80,000 (AUD 162,561) / QALY gained |
| Avancena et al. (2020), USA | (1) FDA-approved Medication-assisted therapies (MATS) (acamprosate and naltrexone) (2) non-FDA-approved MATS (baclofen, gabapentin, topiramate) (3) Counselling (4) do nothing | Healthcare and societal perspective, Lifetime | US Dollars ($); Ref year: 2017 Discount rates: 3% | OWSA (variation in transition probability, cost input and health utility):  Healthcare Perspective:  -All three interventions remained cost-saving with positive NMB using both $50,000 and $100,000 WTP when compared to do nothing.  -when treatment effectiveness of FDA-approved MATS decreased by 24% = ICER exceed $100,000/QALY threshold.  -when treatment effectiveness of non-FDA-approved MATS decreased by 17% and 9% = ICER exceeds $100,000/QALY threshold  Societal Perspective:  - probability of death exceeded 0.033 = FDA-approved MATS not cost-saving  - age of patient exceeded 61= FDA-approved MATS not cost-saving  Scenario Analysis (alcohol treatment costs applied each year over 5 and 10 years):  Healthcare Perspective:  -all three interventions were cost-saving when compared to do nothing  PSA (all parameters varied simultaneously):  Healthcare Perspective:  -all three interventions were cost-saving when compared to do nothing  Societal Perspective:  -FDA-approved MATS vs Do nothing = cost-saving  -Non-FDA-approved MATS vs Do nothing = cost-saving  -Counselling vs Do nothing = $9,213/QALY (AUD 14,696)  PSA (across different age cohorts):  Healthcare Perspective:  -all three interventions were cost-saving when compared to do nothing  Societal Perspective:  -Age 25, 35 and 45 = all three interventions were cost-saving when compared to Do nothing  -Age 65  FDA-approved MATS= $5,490/QALY (AUD 8,757)  Non-FDA-approved MATS= $13,101/QALY (AUD 20,898)  Counselling = $29,863/QALY (AUD 47,637)  WTP: $50,000 (AUD 79,758) and $100,000 (AUD 159,517) /QALY gained |

Note: PSA: probabilistic sensitivity analysis. OWSA: one-way sensitivity analysis. WTP: willingness-to-pay threshold. DSA: deterministic sensitivity analysis.
